# Differences in Cold and Hot Decision-Making between Gambling and Other Addictions

**DOI:** 10.3390/bs14050365

**Published:** 2024-04-25

**Authors:** Sara Meca, Francisco Molins, Maragda Puigcerver, Miguel Ángel Serrano

**Affiliations:** Department of Psychobiology, Universitat de València, Avenida Blasco Ibañez, 21, 46010 Valencia, Spain; sameza@alumni.uv.es (S.M.); francisco.molins@uv.es (F.M.); mapuigp2@uv.es (M.P.)

**Keywords:** gambling, biological addictions, decision-making, cold decisions, hot decisions, IGT

## Abstract

Behavioral and biological addictions can impair decision-making processes, mainly by means of a dysfunction in brain regions associated with reward and frontal areas that may lead to disadvantageous choices. Understanding these differences helps establish appropriate terminology and enhances our ability to recognize, prevent, and treat these disorders effectively. Thus, while behavioral and biological addictions share some common elements, their underlying mechanisms and impact on decision-making vary significantly. Moreover, decision-making can be measured through questionnaires (stable or “cold” measures) or dynamic tasks (hot decisions) such as the Iowa Gambling Task (IGT), which can reflect different dimensions of this process. The aim of this study was to compare decision-making from different perspectives—stable and dynamic measures—in patients with gambling addiction (GA) (*n* = 42) and patients with biological addictions (BA) (n = 43). Decision-making was assessed using GDMS (Decisional Styles) and the LCT (Loss Aversion), as cold decision-making measures, as well as a hot or situational task called the IGT (Iowa Gambling Task). The results revealed that GA patients exhibited lower rational style scores compared to BA patients. Additionally, GA patients showed greater loss aversion according to the LCT questionnaire. On the other hand, when analyzing the IGT results, no differences were observed between groups in the overall IG index, learning curves, or the loss aversion parameter. However, GA patients showed higher sensitivity to feedback and less consistency in their decisions. These findings highlight the differences between different types of addictions and highlight the importance of considering the type of measure used to evaluate decision-making.

## 1. Introduction

Behavioral and biological addictions can profoundly influence decision-making processes, albeit through distinct mechanisms [1]. Behavioral addiction manifests when individuals develop compulsive patterns of engagement with specific behaviors or activities, distinct from substance addiction, which entails the misuse of substances such as drugs or alcohol. In contrast to substance addiction, characterized by the consumption of psychoactive substances, behavioral addiction pertains to repetitive engagement in activities such as gaming, gambling, or compulsive buying [2]. These divergent manifestations of addiction underscore the multifaceted nature of addictive disorders, necessitating tailored approaches to intervention and treatment [3]. Both pathological gambling and substance use disorders share genetic risks for impulsivity and reward-seeking [4]. Moreover, research over the last two decades indicates that drug addiction and gambling act in similar ways on the brain, mainly by means of a dysfunction in regions associated with reward and frontal areas that may lead to disadvantageous choices [2,4]. Hence, it has been hypothesized that impairments in decision processes lie at the core of addictive disorders [5]. However, while behavioral and biological addictions share some common elements, their underlying mechanisms and impact on decision-making vary significantly [6]. Understanding differences could help to establish appropriate terminology and would enhance our ability to recognize, prevent, and treat these disorders effectively.

Gambling is a naturalistic example of risky decision-making [5]. Excessive gambling involvement (i.e., pathological gambling) is currently conceptualized as a behavioral addiction and its neuropsychological basis are being studied extensively [7]. Thus, most researchers characterize excessive gamblers as demonstrating cognitive distortions in their core belief systems about their ability to win at gambling [2,8,9]. In fact, cognitive factors such as risk-taking and decision-making are intrinsically related to the addictive behavior itself [6,10,11]. Moreover, disadvantageous decision-making and increased risk-taking may lead to problematic behaviors such as substance use and abuse, pathological gambling, and excessive internet use [12].

In the case of biological addictions, chronic use of substances is associated with neural dysfunctions and related cognitive deficits that affect brain regions responsible for emotional regulation and cognitive processes [13], including decision-making [5]. Altered cognitive function can be viewed as a frame of substance use disorders, with alterations in executive functions (attention, inhibition/regulation, working memory, and decision-making). Thus, poor cognitive (sometimes referred to as “top-down”) regulation motivational processes—whether appetitive (reward, incentive salience) or aversive (stress, negative affect)—are recognized as a fundamental impairment in addiction and a potentially important target for intervention [5,14].

In both cases, the ventral tegmental area—nucleus accumbens—and the orbital frontal cortex circuit that process incoming reward inputs are impaired [6]. These brain areas are related to decision-making. In fact, decision-making could be the key feature of understanding behavioral and biological addictions. Decision-making is a complex set of cognitive processes that enables individuals to choose the most optimal course of action after reasoned consideration of existing alternatives. The assessment of decision-making in addictive disorders primarily centers around performance-based neurocognitive tasks, often compared to other cognitive variables such as executive functions or intelligence. Individuals affected by addictive disorders and/or gambling disorder exhibit similar features across various decision-making tasks [15], as has been shown with the Iowa Gambling Task (IGT), the leading tool used to measure decision-making [6]. Individuals affected by addictive disorders and/or gambling disorder exhibit similar features across various decision-making tasks, although there is no consensus within the scientific literature regarding the obtained results. Numerous studies have explored the intersection between addictive disorders and decision-making abilities, revealing a complex and heterogeneous landscape marked by divergent findings and interpretations. Some research suggests that individuals with addictive disorders may exhibit impaired decision-making capabilities, characterized by heightened impulsivity, risk-taking propensity, and diminished sensitivity to reward and punishment cues [16,17]. Such deficits are theorized to underpin maladaptive behaviors observed in addiction, such as compulsive substance use or excessive gambling. Conversely, conflicting evidence also exists, with certain studies failing to identify significant differences in decision-making performance between individuals with addictive disorders and healthy controls [18,19]. These discrepancies may stem from methodological variations across studies, including differences in sample characteristics, task paradigms, and the measurement tools employed. Despite these inconsistencies, the recognition of shared features in decision-making among individuals with addictive disorders underscores the need for targeted interventions aimed at addressing the cognitive vulnerabilities associated with addiction. Interventions targeting decision-making processes may encompass cognitive behavioral therapies, mindfulness-based approaches, and pharmacological interventions designed to mitigate impulsivity and enhance self-regulatory capacities [20]. Therefore, it is necessary to delve deeper into how decision-making is measured to resolve these inconsistencies. From our perspective, two aspects may be relevant: the way decision-making is measured and the need to use mathematical models to study the underlying processes of decision-making.

Decision-making can be measured through questionnaires (stable measures) or dynamic tasks such as IGT, which can reflect different dimensions of this process [21]. In fact, decision context is a primary variable to understand decision-making in gamblers [22]. Psychometric tests or questionnaires are considered to assess “cold” neurocognitive measures of executive functions (EFs) in adults with a substance use disorder, considering that people respond in an aseptic situation. However, hot decision-making processes could be related to dynamic tasks, where participants make decisions depending on the task development. These “hot” decisions could be associated with emotional, affective, and visceral responses, while cold executive functions could be associated with rational decision-making [23]. In this sense, in a single study, the IGT failed to distinguish between patients with polysubstance abuse and controls, and it was not linked to social adjustment, while BRIEF-A, a test that allows evaluating executive functions in adult population, did [24].

Another point to highlight is that the IGT has traditionally been used to assess overall performance, understanding decision-making as a unidimensional construct, with the IG score being used to classify people as good or bad decision makers. Moreover, a learning curve can be obtained that allows tracking of the learning curve over time. However, current research asserts that decision-making is not a singular entity [25]. Instead, it involves multiple components [26]. Bayesian cognitive modeling is a statistical method that allows the decomposition of the IGT into different components and the identification of specific deficits, such as feedback sensitivity, loss aversion, choice consistency, or learning processes. All of these processes underlie decision-making and could be a key factor to understanding differences in biological and behavioral decision-making.

Therefore, the aim of this study was to explore the differences in decision-making in people with biological and gambling addictions, using “cold” and “hot” measures (adding computational models) to test whether the differences found in the literature could be explained by these factors.

## 2. Materials and Methods

### 2.1. Participants

Participants were recruited from a private therapy center specializing in addictions during 2022–2023. The study comprised a total of 85 patients, categorized according to their specific diagnoses into two groups: pathological gambling, with 42 participants (age: M = 36.38, SD = 11.63; women: 92.86%), and biological addictions (alcohol, cocaine, etc.), with 43 participants (age: M = 40.08, SD = 12.57; women: 70.73%). All of them were evaluated by specialized clinical psychologists to verify that they met the diagnostic criteria for each disorder according to the Diagnostic and Statistical Manual of Mental Disorders, fifth version (DSM-5) [27].

### 2.2. Procedure

This was an observational study in which two groups were compared in terms of their decision-making. All participants in this study were enrolled in an addiction clinic. At this specialized clinic, they underwent comprehensive evaluations to assess the severity and nature of their addictive behaviors. These assessments were crucial for identifying individuals with addiction-related issues. Once the presence of addictive problems was established, these individuals were cordially invited to actively participate in the study. Both groups read and signed informed consent and completed the first battery of questionnaires, which included socio-economic questions, such as age, gender, and educational level. To verify that the pathological gambling group was well diagnosed and differed from the group with biological addictions, both groups were subjected to the National Opinion Research Center DSM-IV Screen for Gambling Problems (NODS) questionnaire, which addresses the severity of pathological gambling. Moreover, to assess the general tendency (trait dimension) of decision-making, the General Decision-Making Styles (GDMS) and the Lottery Choice Task (LCT) questionnaires were used. On the other hand, to assess in situ decisional ability (state dimension), both groups performed the computerized version of the IGT. This study was approved by the Ethics Research Committee of the University of Valencia in accordance with the ethical standards of the 1969 Declaration of Helsinki (Number: H1543999098237).

#### 2.2.1. National Opinion Research Center DSM-IV Screen for Gambling Problems

The NODS [28] is a well-established diagnostic tool used to assess pathological gambling based on the criteria of the DSM-IV. It consists of 17 items that inquire about lifetime and past-year gambling behaviors and problems. Each item corresponds to a specific DSM-IV criterion for pathological gambling. The NODS was conducted through an interview, providing a comprehensive evaluation of the gambling behaviors and symptoms. The scores range from 0 to 17, with higher scores indicating a greater presence and severity of gambling-related issues. The NODS is widely recognized for its reliability and validity in both clinical and research settings, offering a thorough assessment of the extent and impact of gambling behaviors.

#### 2.2.2. General Decision-Making Styles

The GDMS survey, adapted from Scott and Bruce [29], consists of 24 statements across five scales: rational, intuitive, dependent, avoidant, and spontaneous. Participants rate their agreement on a scale from 1 (“strongly disagree”) to 5 (“strongly agree”). This instrument assesses different decision-making approaches, such as logical (rational), gut-feeling (intuitive), seeking others’ input (dependent), delaying decisions (avoidant), and quick choices (spontaneous). The reliabilities were rational (0.85), intuitive (0.84), dependent (0.86), avoidant (0.94), and spontaneous (0.87) [29].

#### 2.2.3. Lottery Choice Task

An ad hoc Spanish translation of the Lottery Choice Task (LTC) [30] was employed. In this task, participants had to decide along six lotteries whether they would accept or reject the bet. In each lottery, the gain was fixed at EUR 6 and the loss varied through bets (ranging from EUR 2 to 7), yielding a successively decreasing expected value for each lottery. This instrument identifies the participants’ level of loss aversion. Following Hadlaczky et al. [31], loss aversion is defined as the inverse of the highest accepted gamble, thus providing a continuous variable ranging from 0 to 6, where higher scores indicate higher loss aversion, since the ratio gains/losses would be higher. This ratio would show how big the potential gain must be in relation to the potential loss for the bet to be accepted.

#### 2.2.4. Iowa Gambling Task

Decision-making was appraised using the computerized version of IGT [32]. Participants aimed to maximize their returns across 100 sequential choices, with opportunities to earn or lose money. They had the option to select from four card decks: two being disadvantageous (A and B) and two advantageous (C and D). Decks A and B yield substantial immediate rewards but incur greater future losses. Conversely, C and D offer modest short-term rewards but entail smaller long-term losses, leading to enhanced profitability. Following each choice, the participants received feedback, aiding in refining subsequent decisions. Performance was gauged by the IG index: the count of C and D picks minus the count of A and B choices. This index was computed for the entire task (IG Total) and for 20-trial segments to analyze the learning trajectory. Moreover, the PVL model was employed to decipher the processes steering reinforcement learning in the IGT.

#### 2.2.5. Prospect-Valence Learning Model

The Prospect Valence Learning (PVL) model incorporating the Delta learning rule (PVL-Delta) [33] was utilized to deduce 4 parameters. The first was feedback sensitivity (α), ranging from 0 to 1, where values closer to 1 denote that the subjective utility is amplified, meaning task execution is influenced by the scale of gains and losses; smaller values suggest that all gains and losses are perceived equivalently, irrespective of magnitude, and therefore the frequency of gains and losses is pivotal rather than their magnitude. The second was loss aversion (λ), with a range from 0 to 5, where 1 signifies equal sensitivity to losses and gains; values below 1 indicate heightened sensitivity to gains, and values above 1 reflect increased sensitivity to losses, denoting loss aversion. Thirdly, learning (A), spanning from 0 to 1, reflects the emphasis a participant places on prior deck interactions versus the most recent outcome. A higher A value suggests a stronger impact of the latest card on deck expectations, indicating rapid disregard of prior choices. Conversely, a lower value signifies a dominance of past experiences, meaning enhanced learning. Lastly, the consistency (c) parameter, varying from 0 to 5, denotes whether choices align with expectations or are made randomly. A value nearing 0 implies randomness, whereas a higher value indicates a stronger alignment with expectations.

Each parameter of the PVL-Delta model was estimated for each participant through Hierarchical Bayesian Analysis (HBA) [33], performed with the hBayesDM package for the R software. The hBayesDM uses Stan 2.1.1 [34] with the Hamiltonian Monte Carlo (HMC) algorithm as MCMC for sampling the posterior distributions. Following Alacreu-Crespo et al. [25], we drew 40,000 samples, after a burn-in of 23,333 samples, in three different chains (in sum, a total of 120,000 samples and 70,000 burn-ins). The Gelman–Rubin test [35] was used to determine whether the chains converged (Ȓ) to the target distribution. The Ȓ values of all parameters were 1, which means that convergence was achieved. In addition, to confirm this convergence, the MCMC chains were visually inspected. 

### 2.3. Statistical Analyses

Outliers were identified using the 2.5 standard deviations method. Normality was assessed using Kolmogorov–Smirnoff with Lilliefors correction. The principal analyses consisted of multivariate general linear models [specifically multivariate analysis of variance (MANOVA)] that allowed for the examination of differences between two groups (pathological gambling versus biological addictions) in two main aspects. Firstly, in the severity of pathological gambling according to the NODS (both lifetime and in the past year), and secondly, in the different decision-making variables, both dispositional (GDMS and LTC) and situational (IGT). The significance level (α) was set at 0.05, and the partial eta square (η^2^_p_) indicated the effect size. All analyses were conducted using IBM SPSS Statistics 25.

## 3. Results

### 3.1. Descriptive Statistics

In Table 1, the means and standard deviations of all the variables of interest included in this study are presented. Furthermore, it can be observed that, although both groups were homogeneously distributed considering their age and educational level (*p* > 0.05), the pathological gambling group obtained significantly higher scores on the NODS questionnaire, both throughout their lifetime and in the last year (see Table 1), reflecting the greater severity of pathological gambling behavior in this group and, therefore, distinguishing it from the group with other biological addictions and assuring the right diagnostic.

### 3.2. Dispositional Decision-Making Assessed with GDMS and LCT

**Figure 1:**
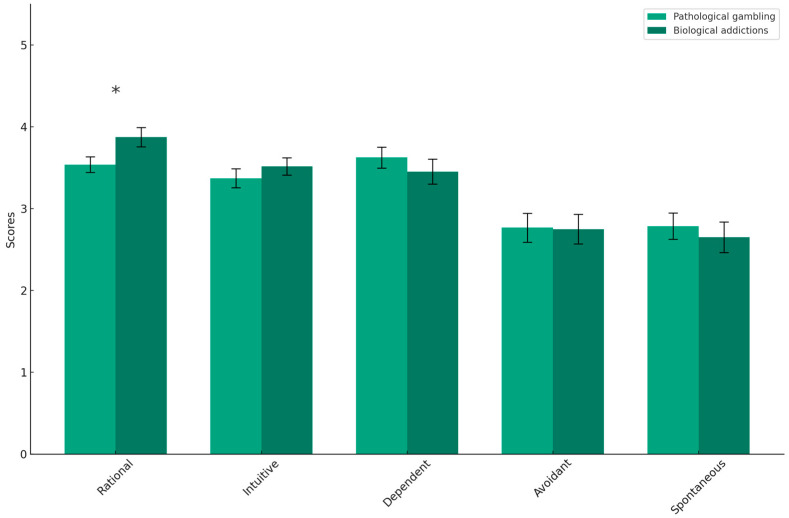
Scores on the GDMS for each of the decisional styles. The asterisk indicates that differences were significant at the 0.05 level.

To evaluate the stable dimension of decision-making, that is, the general tendency when deciding, it was analyzed whether both groups (pathological gambling and biological addictions) differed in any of the decisional styles evaluated with GDMS or in their level of loss aversion extracted with the LCT. As reflected in Table 1 and Figure 1, the MANOVA revealed that, although the scores for most decisional styles were similar in both groups, the pathological gambling group showed a lower tendency to decide logically, with significantly lower scores in the rational decisional style. In line with this result, this same group exhibited more irrational decision-making, being more influenced by the loss aversion bias, with higher scores of this bias in the LOT (see Table 1).

### 3.3. Situational Decision-Making Assessed with IGT

**Figure 2:**
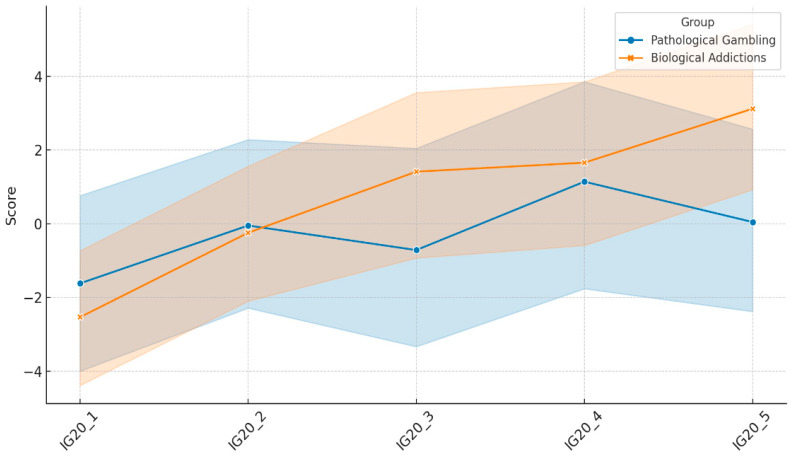
Learning curve in IGT. IG indexes ([decks C + D] − [decks A + B]) for each block of 20 decisions, according to the group. The shaded regions represent the 95% confidence interval.

To assess the more dynamic dimension of decision-making, that is, the decision-making capacity in situ, the prominent IGT was used. As can also be seen in Table 1, both groups (pathological gambling and biological addictions) had similar decision-making capacities, with total averages of −1.19 and 3.41, respectively. Likewise, their learning curves (see Figure 2) did not differ in a statistically significant way, revealing similar learning during the task.

However, delving into the cognitive subprocesses during the IGT, extracted through the PVL Delta model, the MANOVA revealed notable results. On one hand, both groups exhibited similar scores for loss aversion and learning, with the latter being in line with the overall scores and learning curves. However, there were statistically significant differences in sensitivity to the feedback received during the task and in the consistency of their decisions, with higher sensitivity to feedback but lower decisional consistency for the pathological gambling group compared to the biological addictions group (all means and MANOVA statistics can be consulted in Table 1).

## 4. Discussion

The results of our study revealed that, as expected, higher scores on the NODS were observed in pathological gamblers compared to in patients with other addictions. However, concerning decision-making variables, we observed distinct profiles within each group. Specifically, in the questionnaire-based assessments, pathological gamblers exhibited lower scores in rational decision-making style and greater aversion to losses. Regarding decision-making assessed through the IGT, while no significant differences emerged in basic indicators (IG index and learning curve), we did observe heightened sensitivity to feedback and lower consistency among gamblers compared to patients with other addictions. Thus, our findings highlight differences in both “cold” and “hot” decision-making processes.

### 4.1. Cold Decision-Making

In relation to the questionnaires (representing “cold” decision-making), the fact that pathological gamblers exhibit less rationality in their decision-making styles (with no differences in other styles) may reflect a “warmer” decision-making—choices made with a tendency to more emotional involvement. This could represent an anomaly that might serve as the starting point for the impulsivity commonly associated with pathological gambling [36,37]. Complementarily, the higher aversion to losses, as measured by the Lottery Choice Task questionnaire, aligns with findings from the existing literature [5,22], and reflect more biased, irrational decisions [38]. Participants with biological addictions also showed loss aversion (lambda = 1.86), but at a level similar to that indicated by the literature in healthy populations, where losses weighed twice as much as gains (lambda = 2) [39]; however, those participants involved in pathological gambling showed a significantly higher level of loss aversion, representing that losses would weigh approximately three times as much as gains (lambda = 3.06). This could reflect that, in pathological gamblers, at least in “cold” situations, an impact bias occurs, whereby the negative impact of potential losses is overestimated [40], leading to a greater emotional response, even in these situations, associated with less rational decision-making. When combined, these two results suggest that the decision-making process in pathological gamblers is affected. Despite their greater aversion to losses, their less rational decision-making style may hinder more conservative behaviors, ultimately leading to a reduction in gambling behavior. Consequently, these questionnaires may reveal a decision-making trait that allows for the establishment of profiles for the early detection of pathological gamblers.

### 4.2. Hot Decision-Making

On the other hand, regarding the dispositional or “hot” decision-making assessed with the IGT, there were no differences in the overall IG index, learning curves (which are generally poor), or the computational parameter related to loss aversion, with the latter demonstrating that these patients do not actually suffer a greater negative impact or “pain” from losses [41] compared to gains when they actually lose, as opposed to their overestimation of potential losses when evaluated cold. However, patients with pathological gambling behaviors exhibited greater sensitivity to the overall feedback (IGT_alpha_sensitivity_feedback). In other words, both gains and losses (and not only losses) had a stronger impact and served as stronger “rewards” or “punishments” in the pathological gambling group compared to those with other addictions. This heightened impact is likely due to the nature of the disorder itself: for individuals addicted to gambling, winning or losing holds greater significance. Moreover, as discussed, pathological gamblers exhibit dysfunctions in their salience network, and, more specifically, within their reward systems [5], key regions in the processing of both gains and losses, which could be responsible for the magnified impact of both stimuli. Additionally, these patients also exhibit deficits in their prefrontal cortex, crucial in evaluating the magnitude of gains and losses and their subsequent processing for logical or rational decision-making. This may explain why, despite receiving more reinforcement or punishment, according to the “Alpha” parameter, we observed that they also exhibited lower decision consistency based on the “IGT_C_consistency” parameter. As Damasio proposed, accurately capturing and processing emotional feedback following outcomes (punishments and rewards) is crucial in IGT, where information is not as explicit and must be acquired through experience while playing. This ability to learn from feedback and act consistently ultimately leads to greater decision-making consistency. Thus, it could be possible that gamblers’ decisions lack a stable pattern and appear more random, lacking a clear direction. Therefore, these findings highlight the intricate interplay between feedback sensitivity, reinforcement, and decision consistency in pathological gamblers.

Therefore, these nuanced variations underscore the need for tailored future interventions and diagnostic refinement in the context of addictive disorders, differentiating between biological addictions and gambling. Understanding decision-making profiles may serve as a valuable tool for targeted therapeutic approaches and improved clinical outcomes.

### 4.3. Limitations

There are some limitations that have to be taken into account in order to interpret the results. The primary limitation lies in the small sample size within each group, particularly in the biological addictions group, where differentiation among specific addictions was not feasible. Moreover, another important limitation is the absence of a control group without addiction. Future studies should more comprehensively analyze decision-making differences across various addictions. In addition, another limitation is the use of statistical testing without the prevention of type 1 errors, which could affect the reliability of the results. Another limitation is that it is a cross-sectional study, which does not allow for conclusions that can predict decisional behavior. Lastly, considering that there may be brain-level differences underlying the observed variations, the study’s limitation lies in not measuring physiological variables that could directly or indirectly reflect the assessed decision-making processes.

## 5. Conclusions

In conclusion, our study highlights the nuanced differences in the decision-making processes between pathological gamblers and individuals with other addictions. While both groups showed higher NODS scores, as expected, we observed distinct profiles within each group in relation to rational decision-making style, aversion to losses, and decision-making assessed through the IGT. The findings suggest that pathological gamblers exhibit a less rational decision-making style, higher aversion to losses, and greater sensitivity to feedback, but lower decision consistency compared to those with other addictions. This intricate interplay between feedback sensitivity, reinforcement, and decision consistency underscores the need for tailored interventions and diagnostic refinement in the context of addictive disorders, differentiating between biological addictions and gambling. Ultimately, understanding decision-making profiles may serve as a valuable tool for targeted therapeutic approaches and improved clinical outcomes.

## Figures and Tables

**Table 1:** Descriptive statistics and ANOVAs for the assessed variables.

	Pathological Gambling (N = 42)	Biological Addictions (N = 43)	F	*p*-Value	η^2^_p_
Age	36.38 ± 11.63	39.85 ± 12.33	1.74	0.190	0.021
Educational level	4.95 ± 2.32	4.58 ± 2.25	0.55	0.462	0.007
NODS lifetime	13.34 ± 2.95	2.52 ± 4.77	153.43 ***	<0.001	0.654
NODS last year	8.90 ± 6.21	1.95 ± 4.27	35.45 ***	<0.001	0.304
GDMS Rational	3.57 ± 0.60	3.86 ± 0.80	4.95 *	0.028	0.043
GDMS Intuitive	3.40 ± 0.76	3.54 ± 0.70	0.79	0.378	0.010
GDMS Dependent	3.62 ± 0.81	3.48 ± 0.96	0.49	0.488	0.006
GDMS Avoidant	2.70 ± 1.12	2.73 ± 1.15	0.01	0.922	0.000
GDMS Spontaneous	2.81 ± 1.06	2.66 ± 1.20	0.32	0.572	0.004
LOT Loss aversion	3.06 ± 2.23	1.86 ± 1.62	6.82 *	0.011	0.088
IG TOTAL	−1.19 ± 25.03	3.41 ± 16.45	0.98	0.326	0.012
IG20_1	−1.62 ± 8.36	−2.54 ± 5.85	0.33	0.565	0.004
IG20_2	−0.05 ± 7.86	−0.24 ± 6.05	0.02	0.899	0.000
IG20_3	−0.71 ± 8.82	1.41 ± 7.35	1.42	0.236	0.017
IG20_4	1.14 ± 9.86	1.66 ± 7.69	0.07	0.792	0.001
IG20_5	0.05 ± 8.46	3.12 ± 7.99	2.90	0.093	0.035
Learning (A)	0.33 ± 0.15	0.35 ± 0.27	0.17	0.683	0.002
Feedback sensitivity (α)	0.28 ± 0.09	0.24 ± 0.06	6.11 *	0.015	0.069
Consistency (c)	0.65 ± 0.31	0.78 ± 0.15	6.51 *	0.013	0.073
Loss aversion (λ)	0.39 ± 0.46	0.47 ± 0.49	0.70	0.405	0.008

*M*ean ± *SD*; *** significant contrast at the 0.001 level; * significant contrast at the 0.05 level.

## Data Availability

Data will be provided upon request to M.A.S. (m.angel.serrano@uv.es).
